# Polyacrylic acid-coated iron oxide nanoparticles could be a useful tool for tracking inflammatory monocytes

**DOI:** 10.2144/fsoa-2019-0066

**Published:** 2019-10-30

**Authors:** Manuela Giraldo-Villegas, Jeaneth Urquijo, Oscar L Arnache-Olmos, Mauricio Rojas-López

**Affiliations:** 1 Grupo de Inmunología Celular e Inmunogenética, Sede de Investigación Universitaria (SIU), Universidad de Antioquia (UDEA), Calle 70 No. 52-21, Medellín, Colombia; 2 Grupo de Física del Estado Sólido, Sede de Investigación Universitaria (SIU), Universidad de Antioquia (UDEA), Medellín, Colombia; 3 Unidad de Citometría de Flujo, Sede de Investigación Universitaria (SIU), Universidad de Antioquia (UDEA), Medellín, Colombia

**Keywords:** cytokines, inflammation, iron oxide nanoparticles, monocyte-derived macrophages, monocytes, mononuclear phagocytes, nanoparticles, nonclassical monocytes

## Abstract

**Aim:**

To establish the effect of poly(acrylic acid)-coated iron oxide nanoparticles (PAC-IONs) and later exposure to a magnetic field on the differentiation of mononuclear phagocytes into macrophages.

**Methods:**

By flow cytometry, cell death was evaluated with DIOC6 and PI, Poly (ADP-ribose) Polymerases (PARP) fragmentation, H2AX phosphorylation and TUNEL assay. Cytokines by Cytokine bead array and the intracellular amount of iron by atomic absorption spectrometry.

**Results:**

PAC-IONs did not induce apoptosis, modify the cell membrane integrity or alter the mitochondrial membrane potential. They did not affect the cell morphology, the pattern of cytokine accumulation or the activating role of differentiation of mononuclear phagocytes into macrophages on the proliferation of autologous T cells.

**Conclusion:**

This evidence indicates that the PAC-IONs are safe and biocompatible. Moreover, the selectivity of the PAC-IONs for mononuclear phagocytes, as well as their increased uptake by non-classical monocytes, warrant future research with a view to their use as a contrast agent, a useful tool for in vivo tracking of tissue-infiltrating mononuclear phagocytes.

## Lay abstract

In the search for materials that allow the study of inflammatory processes when biopsies are not feasible, magnetic nanoparticles have become an alternative tool for use in MRI. This article examined whether supermagnetic iron nanoparticles can affect the basic function of phagocytic cells, with a view to their use in clinical imaging applications.

## Graphical abstract

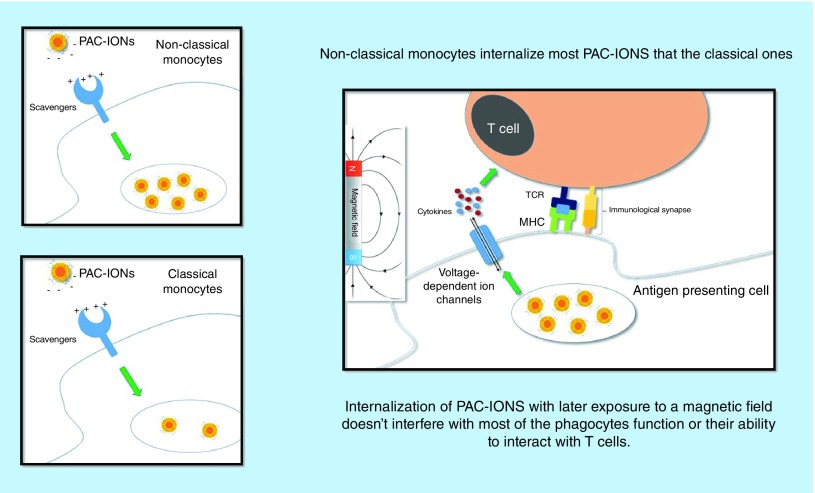

The mononuclear phagocyte (MP) system plays essential roles in the maintenance of tissue homeostasis during the steady state, in the orchestration and genesis of the adaptive immune response, as well as in inflammation and its resolution [1–4]. Circulating monocytes represent a versatile and dynamic cell population [5,6]. In humans, the monocyte subpopulations were conventionally defined based on the surface expression of CD14 and CD16 [7]; classical (CD14^+^CD16^-^) monocytes constitute around 85% of the circulating pool of monocytes, while the remaining 15% consist of intermediate (CD14^+^CD16^+^) and nonclassical (CD14^+^CD16^++^) monocytes [8,9]. Classical monocytes are rapidly recruited to sites of infection and inflammation [10–12], where they exhibit considerable functional plasticity [13]. Over time, classical monocytes become nonclassical ones, and some of them can differentiate into dendritic cells [14–16]. However, during those pathological conditions, tissue damage is only evident at very late phases of the process, when medical intervention is fundamentally palliative. The early detection of monocytes in the tissues could favor the prognosis and intervention of patients in a targeted manner.

Our group has studied monocyte subsets in tuberculosis [17,18] and systemic lupus erythematous [19], including their presence in renal lesions [20]. Altered monocyte counts and functions are observed in several chronic inflammatory processes including childhood obesity [21], diabetes [22,23], atherosclerosis [24–26], cardiovascular diseases [27], infection with HIV [28], Crohn's disease [29] and aging [30], highlighting the diverse roles of monocytes in physiological conditions.

At the early stages of many of these diseases, when the type of tissue damage is not evident, it is difficult to make a prognosis, and any selective intervention on monocytes is not feasible. In many cases, biopsies or bronchoalveolar lavages are required to access the compromised organ; these procedures are invasive and cannot be repeated because of the health risks for patients [31]. Besides, in some cases, the tissue seems to be inaccessible, or it is not possible to detect monocytes due to the systemic compromise. These are the reasons why new tools for tracking monocytes, and even for distinguishing monocyte subsets, are required.

Iron oxide nanoparticles (IONs) have been broadly studied in nanomedicine because of their high biocompatibility [32]. Sugar-coated IONs have been clinically used as contrast agents for MRI in Japan and Europe [33,34]. Human monocytes have been studied with positive contrast agents and superparamagnetic nanoparticles by MRI in noninvasive studies of pathological mechanisms [35–37]. *Ex vivo*-labeled human cells can be successfully reintroduced into patients and tracked by MRI [38]. In the case of some chronic inflammatory diseases, when taking a sample requires an invasive procedure, specific *in vivo* tracking of monocytes will be useful to characterize different patterns of mononuclear infiltrates. *In vivo* studies in 8-week-old male CD-1 mice showed that after intravenous injection, poly(acrylic acid)-coated iron oxide nanoparticles (PAC-IONs) accumulated mainly in the liver and spleen, and at a lower extent in the lungs, without causing severe organ damage [39].

We hypothesized that the PAC-IONs can interact selectively with MPs without affecting their differentiation into mature macrophages (MDMs) and that the subsequent exposure of these cells to a magnetic field (MF) would not induce cell damage or compromise their function as antigen-presenting cells, in terms of cytokine synthesis and induction of activation and proliferation of T cells in response to a mitogen or a conventional antigen. To this purpose, we determined the effects of the PAC-IONs on the differentiation of MPs into macrophages; also, we evaluated the effects of an MF on the ability of those MDMs for activating T cells in response to phytohemagglutinin (PHA) and tetanus toxoid (TT). We also evaluated whether nonclassical and classical monocytes differed in their ability to uptake the PAC-IONs.

## Materials & methods

### Materials

FeCl_2_.4H_2_O, FeCl_3_.6H_2_O, sodium polyacrylate, Histopaque^®^-1077 (1.077 g/ml) and phytohemagglutinin M (PHA-M) were purchased from Sigma-Aldrich (MO, USA). RPMI1640 + GlutaMAX™, penicillin and streptomycin, fetal calf serum and phosphate-buffered saline (PBS) were obtained from GIBCO (Life Technologies, NY, USA). Tetanus toxoid (TT) from *Clostridium tetani* was acquired from Aventis Pasteur (Lyon, France). Molecular-weight cutoff (100 kDa MWCO) cellulose membranes were purchased from Synder Filtration (CA, USA). The cytometric bead array (CBA) for human inflammatory and Th1/Th2 Cytokine Kits, the Apoptosis, DNA Damage and Cell Proliferation Kit, DAPI solution, mouse anti-BrdU-PerCP-Cy™ 5.5 (Clone: 3D4) monoclonal antibody (mAb) and the following mouse antihuman fluorochrome-conjugated mAbs: CD45-PE-Cy7 (Clone: HI30), CD3-PE (Clone: OKT3), CD19-Alexa Fluor^®^ 488 (Clone: HIB19), CD16-BV421 (Clone: 3G8), CD56-BV510 (Clone: NCAM16.2), HLA-DR-FITC (Clone: G46-6), cleaved PARP (Asp214)-FITC (Clone: F21-852), H2AX (pS139)-Alexa Fluor 488 (Clone: N1-431) were purchased from BD Pharmingen™ (CA, USA). Opty Lyse Buffer, and mouse anti-human CD14-PE and CD14-FITC (Clone: 322A-1 [MY4]) mAbs were from Beckman Coulter Inc. (CA, USA). The RosetteSep™ Human Monocyte, T- and B-cell Enrichment Cocktail Kits were obtained from STEMCELL Technologies (Vancouver, Canada), and Polymorphprep™ from Abbott Diagnostics Technologies AS (Oslo, Norway). Carboxyfluorescein diacetate succinimidyl ester (CFSE), DIOC_6_, 7-AAD and propidium iodide (PI) were purchased from Thermo Invitrogen (MA, USA), and Bicinchoninic Acid Assay from Merck KGaA (Darmstadt, Germany).

### Synthesis of nanoparticles

PAC-IONs were prepared by the coprecipitation method, according to Lin *et al.* [40] in the ‘Grupo de Estado Sólido of the Instituto de Física at Universidad de Antioquia.’ Briefly, magnetic magnetite–maghemite particles were obtained by coprecipitation from an aqueous alkaline solution of FeCl_2_.4H_2_O and FeCl_3_.6H_2_O (1:2 stoichiometric ratio) in the presence of 0.4% (w/w) sodium polyacrylate as a stabilizing agent. The pH was adjusted to 12 by the automatic addition of 1 M NaOH, using a 907 Titrando (Herisau, Switzerland). Previous to the synthesis procedure, solutions were passed under an N_2_ (g) flow. During the synthesis, the N_2_ (g) flow was kept constant to avoid oxidation of the oxide particles after their formation. The precipitate obtained was dialyzed with a Spectra/Por^®^ cellulose membrane (100 kDa MWCO) against type II deionized water, until the conductivity of the washing water was similar to that of the deionized water. An aliquot of the particle suspension was stored at room temperature for *in vitro* analyses, and another one was vacuum dried at room temperature and stored under N_2_ atmosphere for further analysis.

### Nanoparticle characterization

Morphological, physical and chemical characteristics of the PAC-IONs were evaluated by different methods. The hydrodynamic particle diameter, size distribution and zeta potential were measured by dynamic light scattering with Zetasizer equipment (Malvern Panalytical, Almelo, The Netherlands) at room temperature. To this purpose, dried PAC-IONs were resuspended (1 mg/10 ml) in a 50:50 (v/v) ethanol–water mixture for triplicate-run analysis of size distribution. Another aliquot of dried PAC-IONs was resuspended in water (0.5 mg/10 ml) to evaluate the zeta potential using the Smoluchowski equation. Additionally, the ethanol–water suspension was also utilized for analyzing the particle morphology by transmission electron microscopy, using a JEOL 100-CX II microscope (Jeol Ltd, Tokyo, Japan).

The magnetization of the PAC-IONs was determined in a sample of dried particles under an applied MF with a Physical Property Measurement System (Quantum Design, CA, USA), using a Vibrating Sample Magnetometer and -0.5 to 0.5 T scan at room temperature (300°K). A summary of the physical properties of the PAC-IONs is included in Table 1.

**Table 1.** Physical parameters of magnetite/maghemite nanoparticle – size, zeta potential, saturation magnetization and coercive field.

Size (nm)	ZP (mV)	MS (emu/g)	HC (Oe)
12.4	-49.2	68	12

### Isolation of peripheral blood mononuclear cells

Mononuclear cells were obtained from peripheral blood of healthy volunteers aged 20–30 years. Most of them worked in the Sede de Investigación Universitaria at Universidad de Antioquia and signed a written informed consent approved by the ethics committee of the Institute of Medical Research of the Faculty of Medicine at the University of Antioquia. They declared that they were not taking any medication and that they had neither an autoimmune nor active infectious disease.

EDTA-anticoagulated (4 ml) or defibrinated (60 ml) peripheral blood samples were used to isolate peripheral blood mononuclear cells (PBMCs) for *in vitro* cultures and monocytes by plastic adherence, respectively.

PBMCs were isolated by density gradient centrifugation on Histopaque^®^-1077 (1.077 g/ml) at 900 × *g* for 33 min. Cells were suspended in RPMI supplemented with 50 μg/ml penicillin, 50 μg/ml streptomycin and 10% inactivated (56°C for 30 min) serum; inactivated fetal calf serum (iFCS) was used for PBMC cultures (MCa) and inactivated autologous serum for monocyte cultures (MCb). Before culturing, the PBMC viability was determined by trypan blue dye exclusion; it was always higher than 95%.

### Exposure of PBMCs to the PAC-IONs & the MF

One hundred and fifty thousand PBMCs per well were plated onto 96-well round-bottom plates to a final volume of 200 μl of MCa. Cells were incubated for 12 h at 37°C, 5% CO_2_, with or without 32 μg/ml of PAC-IONs, and exposed or not to a 1.5-T MF for 10 min using an Achieva 1.5T Nova Dual MRI Scanner (Philips Medical System, Best, The Netherlands) in the ‘Instituto de Alta Tecnología Médica (IATM) at Hospital San Vicente Fundación.’ Four hours later, cell viability was assessed in terms of plasma membrane integrity and mitochondrial membrane potential. To this purpose, cells were stained with PI (1 µg/ml final concentration) and DIOC_6_ (700 nM final concentration) [41,42], incubated for 20 min at 37°C and 5% CO_2_ and acquired in an LSRFortessa II™ flow cytometer (BD Biosciences, CA, USA).

The potential toxicity of the PAC-IONs and the MF on T-cell proliferation were also evaluated using the Apoptosis, DNA Damage and Cell Proliferation Kit (BD Pharmingen). This kit evaluates the phosphorylation of the histone H2AX on Ser 139 (pS139)-H2AX, the cleavage of PARP1 and the BrdU incorporation as indicators of the type of cell death, DNA damage and cell proliferation, respectively [43,44]. Briefly, PBMCs previously exposed to the PAC-IONs and the MF were stimulated with PHA-M or TT from *Clostridium tetani* for 24 h and pulsed with BrdU for 48 h. Then, cells were labeled with anti-PARP1, anti-(pS139)-H2AX, anti-BrdU antibodies and DAPI for nuclei staining, and acquired in the flow cytometer. The compensation was set by aligning the mean fluorescence intensities of positive and negative events from cell samples stained with every single fluorescent reagent. The positivity for every marker was evaluated using the fluorescence minus one controls.

### PAC-IONs uptake by leukocyte subpopulations

The PAC-IONs uptake by different leukocyte subpopulations was determined by flow cytometry through the evaluation of changes in the cell granularity (Side Scatter (SSC)-A, -H, -W). Total blood (25 μl) was diluted 1:4 with MCa (final volume, 100 μl) and exposed or not to 32 μg/ml of PAC-IONs and incubated for 5.5 h at 37°C and 5% CO_2_. After incubation, cells were stained with 0.5 μl of anti-CD45-PE-Cy7. Erythrocytes were lysed with 300 μl of Opty Lyse Buffer for 10 min and 300 μl of sterile deionized water for an additional 10 min. In some assays, owing to the decreased surface expression of CD14 and CD16 in monocytes, the cells were mixed with the PAC-IONs only for 1.5 h at 37°C; then, the cells were stained with fluorochrome-conjugated mouse antihuman CD45-PECy7, CD14-PE, CD16-BV421, HLA-DR-FITC mAbs to distinguish the monocyte subset interacting with the PAC-IONs. The acquisition was performed in an LSR Fortessa II™ flow cytometer. In the first set of assays, the CD45^+^ cells were subdivided into three regions based on their cell granularity for gating granulocytes, monocytes and lymphocytes. In the second set of assays, monocyte subsets were defined. The cell granularity parameters were analyzed using the Overton subtraction of histograms in FlowJo software version 7.6.2 (Tree Star Inc., OR, USA). The areas under the curves (AUCs) were compared with the Kolmogorov–Smirnov test.

### Isolation of monocytes & differentiation into macrophages

The percentages of CD14^+^ monocytes in the isolated PBMCs were determined by labeling 3.0 × 10^5^ cell/μl with 5 μl of anti-CD14-PE mAb and flow cytometry. Then, 2.5 × 10^5^ or 7.5 × 10^4^ CD14^+^ cells/well were plated in 48- or 96-well dishes at a final volume of 500 and 200 μl, respectively. The cells were cultured in media containing 0.5% FCS, at 37°C and 5% CO_2_, and were allowed to adhere to plastic. Four hours later, the monolayer was washed with PBS containing 0.5% FCS at 37°C to remove nonadherent cells, followed by checking under an inverted microscope [17]. Then, the adherent cells were cultured for 5 days until differentiated into macrophages in the presence or absence of 32 μg/ml of PAC-IONs. The cell purity of the monocyte-derived macrophage monolayers (MDMMs) was determined on the 5th day of differentiation by staining with fluorochrome-conjugated mouse anti-human CD3-PerCP, CD19-Alexa Fluor^®^ 488 and CD56-BV510 mAbs to identify the presence of contaminant T-, B- and NK cells, respectively. In all cases, the cell purity of the MDMMs was higher than 95%.

### Exposure of the MDMMs to the MF

After overnight incubation at 37°C and CO_2_, the MDMMs differentiated in the presence or absence of 32 μg/ml of PAC-IONs were exposed or not to a 1.5-T MF for 10 min with the following parameters: TR = 4200 ms, TE = 102 ms, flip angle of 90°, echo train length of 10, 5-cm field of view, 2-mm section thickness, 0.2-mm intersection gap and 256 × 160 matrix. Then, MDMMs were incubated for 5 days at 37°C and CO_2_. On the 5th day, the supernatants were collected and stored at -20°C for measuring the levels of TNF-α and IL-8, IL-1β, IL-6, IL-10 and IL-12p70. The MDMMs were used for cocultures with autologous CD3^+^ T cells as described below.

### Isolation of CD3^+^ T cells by cell sorting

Autologous PBMCs were isolated from the corresponding donors of the monocytes used to generate the MDMMs. PBMCs were labeled with anti-CD3-PerCP mAb to separate total CD3^+^ T cells using the MoFlo™ XDP Cell Sorter (Beckman Coulter Inc.). The purity and efficiency of sorting were higher than 95 and 90%, respectively. Sorted CD3^+^ T cells were washed once and resuspended in 12 ml of PBS, and stained with CFSE at a final concentration of 0.2 μM. The cell suspension was mixed by inversion and incubated for 20 min at 37°C and 5% CO_2_. Subsequently, 1 ml of iFCS was added to quench the unbound CFSE and cells were incubated for an additional 40 min at 37°C and 5% CO_2_ to allow the diffusion of the unbound CFSE. Afterward, the cells were washed twice with PBS at 1000× *g* for 10 min, the supernatant was discarded and the CFSE^+^ T cells were resuspended in media supplemented with 10% iFCS.

### Cocultures of MDMs & CD3^+^ T cells

As previously described, the MDMs, differentiated in the presence or absence of 32 μg/ml of PAC-IONs and exposed or not to a 1.5-T MF for 10 min, were cocultured with autologous CFSE-labeled CD3^+^ T cells (1:2 ratio; MDMs:T cells) and then stimulated with 2 μg/ml of PHA-M or 50 μg/ml TT. PHA-M was used as a positive control of polyclonal T-cell proliferation and TT for stimulating the proliferation of memory T cells. The cocultures were incubated in darkness for 120 h at 37°C and 5% CO_2_. Then, T cells were resuspended, harvested, labeled with 7-AAD (to exclude dying cells), anti-CD4-PE and anti-CD8-eFluor mAbs, and acquired in an LSRFortessa II™ flow cytometer. Proliferation index, division rates and percentages of dividing CD4^+^ and CD8^+^ T cells were determined using the FlowJo software version 7.6.2. Supernatant aliquots were collected before removing T cells from the cocultures and stored at -20°C for measurement of IL-10, IL-4, IL-2, IL-6, TNF-α and IFN-γ levels using the CBA Human Th1/Th2 Cytokine Kit (BD Pharmingen).

### Cytokine determination

Human inflammatory (IL-8, IL-1β, IL-6, IL-10, TNF-α and IL-12p70) and Th1/Th2 cytokines (IL-10, IL-4, IL-2, IL-6, TNF-α and IFN-γ) were measured using the CBA Human Inflammatory and Th1/Th2 Cytokine Kits (BD Pharmingen), following the manufacturer's instructions.

### Isolation of CD14^++^CD16^-^ & CD14^+^CD16^+^ monocyte subsets

A monocyte-enriched cellular suspension was prepared using the RosetteSep Human Monocyte Enrichment Cocktail Kit (STEMCELL Technologies), according to the manufacturer's instructions. Briefly, 50 ml of defibrinated venous blood from healthy volunteers was mixed with 2.5 ml of tetrameric antibody complexes against CD2, CD56, CD19, CD3, CD8, CD66b and glycophorin A, and incubated for 20 min at room temperature. Then, this blood-labeled sample was diluted in 50 ml of PBS containing 2% of FCS and centrifuged in a Histopaque density (1.077 g/ml) gradient at 1200 × *g* for 20 min at room temperature. The cellular monocyte-enriched interface was recovered and washed with 50 ml of PBS containing 2% iFCS. The monocyte-enriched suspension (purity ≥85%) was labeled with anti-CD45-PE-Cy5, anti-HLA-DR-PerCP, anti-CD14-PE and anti-CD16-FITC mAbs for sorting of the CD45^+^HLA^-^DR^+^CD14^++^CD16^-^ and CD45^+^HLA^-^DR^++^CD14^+^CD16^++^ monocyte subpopulations using a MoFlo XDP cell sorter. The isolated monocyte subsets reached purities from 93–98% and the recovery efficiencies for CD14^++^CD16^-^ and CD14^+^CD16^++^ monocytes were ≥90 and ≥70%, respectively. These monocyte subpopulations were allowed to differentiate to MDMs in the presence of PAC-IONs as previously described. Then, the amount of intracellular iron was determined by atomic absorption spectrometry (AAS).

### Isolation of B- & T cells & granulocytes

RosetteSep Human T- and B-cell Enrichment Cocktail Kits (STEMCELL Technologies) were used according to the manufacturer's instructions. To isolate granulocytes, 5 ml of EDTA-anticoagulated blood was carefully layered onto 5.0 ml of polymorphprep in a 15-ml conic tube and centrifuged at 550 × *g* for 30 min in a swing out rotor at 18–22°C, and deceleration without the break. The suspension was transferred to a 15-ml tube, and the cells were pelleted at approximately 400 g for 10 min at 22°C. Granulocytes were resuspended in culture media for subsequent analysis. The purity of all these cell suspensions was evaluated by flow cytometry using fluorochrome-conjugated mouse anti-human CD3, CD56, CD19, CD14 and CD16 mAbs. Isolated T and B cells and granulocytes were exposed to PAC-IONs as previously described. Then, the amount of intracellular iron was determined by AAS.

### Quantification of iron content by AAS

Cells were washed twice with PBS at 37°C and subjected to acid digestion with 3 ml of nitric acid and boiling before being removed. Samples were allowed to reach room temperature; then, 2.5 μl was transferred to 25-ml volumetric balloons, gauged with distilled water and homogenized by inversion. Afterward, the iron content in the samples was quantified using an Atomic Absorption Spectrometer iCE 3000 Series model Flame autosampler controlled by a Thermo Scientific SOLAAR Software (Thermo Fisher Scientific, MA, USA) in the ‘Laboratorio de Análisis Fisicoquímico at Universidad de Antioquia.’ Five hundred microliters of the cell lysates derived from cultures treated or not with PAC-IONs were neutralized with NaOH before protein quantitation with the bicinchoninic acid assay. This measurement was carried out to verify that the differences in the iron content could not be attributable to variations in the number of cells per well. Therefore, iron content was expressed in relation to the protein content.

### Photographic records of MDMs

Photographic records were obtained in an Eclipse TS 100 inverted microscope, provided with phase-contrast achromatic 40X objectives (Nikon, Tokyo, Japan) using a Digital Sight DS-fi1 camera with TV Lens 0.55X DS (Nikon).

### Analysis of data

All experiments were done at least by triplicate. The paired data were compared with the Wilcoxon test. The effect of the PAC-IONs and/or the MF on the cytokine levels were determined by a two-way analysis of variance (ANOVA) with a Bonferroni post-test. p-values < 0.05 were set for statistical significance. Flow cytometric data were analyzed with the FlowJo software version 7.6.2. Statistical analyses were performed using the Statgraphics™ Centurion^®^ XVI software, version 16.1.8 (StatPoint Technologies Inc., VA, USA) and the GraphPad Prism software, version 6.0 (GraphPad Software Inc., CA, USA).

## Results

### PAC-IONs & MF did not affect either the plasma membrane integrity, or the mitochondrial membrane potential of PBMCs

**Figure 1.**
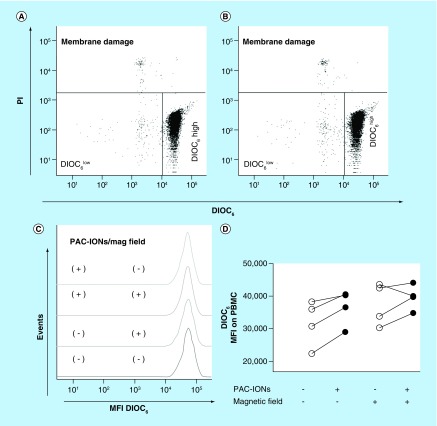
The poly(acrylic acid)-coated iron oxide nanoparticles and the magnetic field do not affect either the cell membrane integrity or the mitochondrial membrane potential of peripheral blood mononuclear cells. Flow cytometer dot plots showing DIOC_6_ and PI staining of PBMCs that were cultured in the absence **(A)** or presence **(B)** of the PAC-IONs. The percentages of DIOC_6_ high (viable), DIOC_6_ low (mitochondrial damage) and PI^+^ (with membrane damage) cells of a representative experiment are shown. The DIOC_6_ MFI of the viable PBMCs was compared by histogram overlay: PBMCs cultured with and without PAC-IONs, and exposed or not to the magnetic field **(C)**. The consolidated values of DIOC_6_ MFI were compared through an analysis of variance, ANOVA II **(D)**; n = 4 independent experiments. ANOVA: Analysis of variance; MFI: Mean fluorescence intensity; PAC-ION: Poly(acrylic acid)-coated iron oxide nanoparticle; PBMC: Peripheral blood mononuclear cell; PI: Propidium iodide.

PBMCs were incubated with 32 μg/ml of PAC-IONs for 12 h and exposed to a 1.5-T MF for 10 min. Initially, the cytotoxic effect of several concentrations of PAC-IONs (from 6.2 to 32 μg iron, quantified by gravimetry) was tested; 32 μg/ml was the higher concentration of PAC-IONs in which cultured cells did not exhibit changes in either the mitochondrial DIOC_6_ uptake or the plasma membrane; these results were similar to previous reports [45]. Viability was evaluated using a DIOC_6_/PI staining. DIOC_6_ detects the reduction of the mitochondrial membrane potential, one of the earliest hallmarks of many different types of cell death; and PI detects cell membrane damage. As shown in Figure 1A & B, the exposure to PAC-IONs did not alter either the proportion of cells with cell membrane damage (PI^+^) or the fraction of DIOC_6_^+^ cells. Furthermore, the different treatments did not change the DIOC_6_ mean fluorescence intensity of viable PBMCs (those with the highest DIOC_6_ uptake; Figure 1C & D). It is important to note that the DIOC_6_ uptake per cell was higher in cultures exposed to PAC-IONs or the MF; however, these effects were not statistically significant. There were no interactions between the PAC-IONs and the MF, according to a two-way ANOVA.

### PAC-IONs &/or the MF did not alter the ability of PBMCs to proliferate, cause DNA damage or increase the signs of cell death

**Figure 2.**
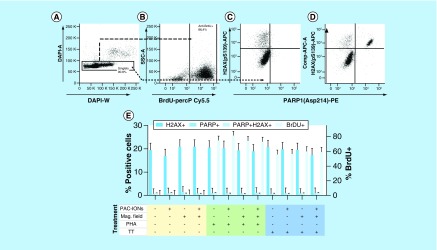
The poly(acrylic acid)-coated iron oxide nanoparticles and the magnetic field do not affect the capacity of peripheral blood mononuclear cells to proliferate and do not induce DNA damage or caspase-3-mediated cell death. **(A)** Flow cytometer dot plots show the strategy for the exclusion of cell aggregates based on DAPI-A versus DAPI-W. **(B)** Definition of BrdU^+^ PBMCs. (pS139) H2AX^+^ and cleaved PARP1^+^ cells in PBMCs treated or not with the PAC-IONs and exposed to the MF **(C)**. Positive control of cell damage: PBMCs were treated with 20 nM Topotecan hydrochloride **(D)**. Consolidated results **(E)** Y1 axis, percentage of (pS139) H2AX^+^ and/or cleaved PARP1^+^ cells. Y2 axis, percentage of BrdU^+^ cells. Comparisons were made with ANOVA II, n = 5 independent experiments. ANOVA: Analysis of variance; MF: Magnetic field; PAC-ION: Poly(acrylic acid)-coated iron oxide nanoparticle; PBMC: Peripheral blood mononuclear cell.

To rule out possible effects of the PAC-IONs on cell proliferation, DNA integrity, or cell viability, PBMCs were exposed to the PAC-IONs and the 1.5 T MF for 10 min; then, PBMCs were stimulated with PHA-M or TT and pulsed with BrdU. Afterward, cells were fixed and permeabilized, labeled with anti-PARP1 (cleaved form), anti-(pS139) H2AX, anti-BrdU mAbs, and DAPI, and acquired in a flow cytometer. The PHA-M stimulus and the BrdU-anti BrdU system evaluate the cell fraction in S (DNA synthesis) phase. Anti-(pS139) H2AX and anti-PARP1 mAbs detect cells with DNA damage and cleavage of PARP1 (a sign of caspase-3-mediated cell death), respectively. Figure 2 shows the strategy used to exclude cellular aggregates (Figure 2A), the definition of BrdU+ (Figure 2B), PARP1^+^ and (pS139) H2AX+ (Figure 2C) cells, and the positive control for DNA damage (Figure 2D). As seen in Figure 2E, the PAC-IONs and/or the MF did not induce significant changes in PBMCs in comparison with the cell controls exposed only to culture media. Besides, PAC-IONs and/or the MF did not alter either the PARP1^+^ or the (pS139) H2AX^+^ cells present in the BrdU^-^ cell fraction (data not shown).

### PAC-IONs are selectively internalized by monocytes

**Figure 3.**
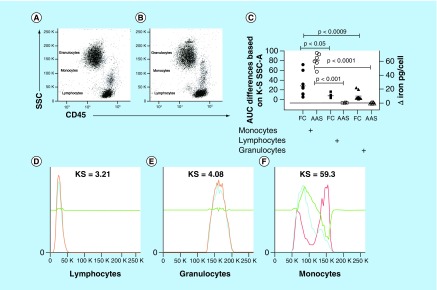
The poly(acrylic acid)-coated iron oxide nanoparticles markedly increase the granularity of peripheral blood monocytes. Whole-blood samples were treated with the PAC-IONs for 5.5 h and stained with anti-CD45-PE-Cy7. Flow cytometer dot plots show granularity versus CD45 to define leukocyte subsets cultured in the absence **(A)** or presence of PAC-IONs **(B)**. **(D–F)** Comparisons of overlaid histograms through the K–S Goodness-of-Fit test in the FlowJo program version 7.6.2. Comparison of the AUCs (left Y-axis, **– C –**) among the three cell populations, according to K–S values. Comparison of the iron content in cells isolated with RosetteSep™ or Polymorphprep™ and then exposed to PAC-IONs for 5.5 h, extensively washed with PBS, counted and lysed to quantify proteins by BCA, and iron by AAS (right Y-axis, **– C –**). In both cases, one-way ANOVA and the Dunn post-test **(C).** n = 10 independent experiments. AAS: Atomic absorption spectrometry; ANOVA: Analysis of variance; AUC: Area under the curve; BCA: Bicinchoninic acid assay; KS: Kolmogorov–Smirnov; PAC-ION: Poly(acrylic acid)-coated iron oxide nanoparticle; PBS: Phosphate-buffered saline; SSC: Side scatter.

Whole blood was exposed to 32 μg/ml of PAC-IONs and incubated for 5.5 h at 37°C. Then, the cells were stained with anti-CD45-PE-Cy7 mAb, and the erythrocytes were lysed. Flow cytometry was used to evaluate changes in cell granularity (SSC), as an indicator of the PAC-ION uptake by the cells. As seen in Figure 3A and B, the SSC versus forward scatter (FSC) dot plots showed obvious changes in the SSC of monocytes. The differences in the PAC-ION uptake by monocytes, lymphocytes, and granulocytes (gated in defined regions) were estimated through comparisons of the AUCs of the SSC histograms for each cell subset from cultures incubated in the presence or absence of PAC-IONs. The Kolmogorov-Smirnov Goodness-of-Fit test showed increased medians for the AUCs of the overlaid SSC histograms between cells from cultures treated with and without PAC-IONs; specifically, the values for monocytes, granulocytes and lymphocytes were 24.55, 6.88 and 3.65%, respectively (Figure 3C).

To confirm that the amount of PAC-IONs internalized by lymphocytes and granulocytes were negligible, blood samples were spliced to isolate T- and B cells and granulocytes. T- and B cells were isolated using RosetteSep Human T- and B-Enrichment Cocktail kits; and, Polymorphprep was used for isolating granulocytes. Cells were counted, and equal numbers were incubated or not with PAC-IONs for 5.5 h. Afterward, cells were extensively washed with PBS for determining the iron content by AAS. The iron content was similar in cells exposed or not to the particles (Figure 3C, right Y-axis); it is important to note that cell concentration and protein content were similar in cultures exposed or not to the nanoparticles. Additional experiments (not shown) were performed to evaluate whether the PAC-IONs could alter the polymorphonuclear neutrophils (PMN) function. These assays showed that the phorbol myristate acetate (PMA)-induced respiratory burst and the mitochondrial stability of PMNs did not change in the presence of PAC-IONs during a 3-h follow-up (data not shown).

### PAC-IONs were internalized by monocytes & remained intracellularly until the differentiation into macrophages was completed

**Figure 4.**
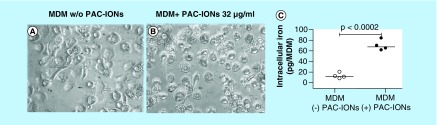
The poly(acrylic acid)-coated iron oxide nanoparticles are internalized by the monocytes and retained intracellularly through their differentiation into macrophages. Photographs were taken with a phase-contrast microscope (40x objective) for MDMs differentiated in the absence **(A)** or presence of PAC-IONs **(B)**. Quantification of the intracellular iron content by AAS **(C)**. The iron picogram-content is expressed as the total value of iron measured divided by the number of cells per well, calculated according to the protein concentration. The comparison was made using a Wilcoxon test for paired samples. p = 0.0002, n = 4 independent experiments with three replicates in each. AAS: Atomic absorption spectrometry; MDM: Differentiation into mature macrophage; PAC-ION: Poly(acrylic acid)-coated iron oxide nanoparticle.

To verify the PAC-ION uptake by monocytes and the intracellular permanence of the particles during the MDM differentiation, monocytes were allowed to differentiate in the absence or presence of 32 μg/ml of PAC-IONs for 5 days and were analyzed by two strategies. The first approach was the analysis of photographic records to compare the morphology of the MDMs. Regardless of the presence of PAC-IONs, the cells were adherent, flattened and showed cytoplasmic projections (Figure 4A & B). The second strategy was the quantification of the cellular iron content in MDMs exposed or not to PAC-IONs. To this purpose, after taking the photos, the monolayers were exhaustively washed to remove the extracellular PAC-IONs; then, the iron content was measured by AAS (Figure 4C). The differences in the cellular iron content allowed to confirm that PAC-IONs were internalized by MPs. Additionally, the cells exposed to the PAC-IONs displayed four- to seven-times more iron content than the non-exposed cells (p = 0.0007 for the ratio between treated and untreated cells; and p = 0.0002 for differences between the respective means).

### PAC-IONs & MF did not affect the MDM viability

**Figure 5.**
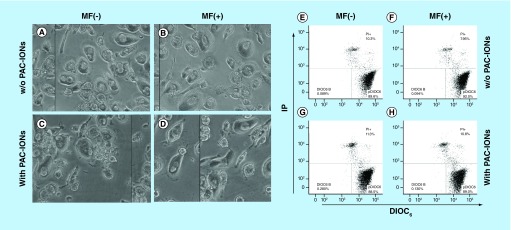
The differentiation into mature macrophages differentiated in the presence of the poly(acrylic acid)-coated iron oxide nanoparticles and exposed to the magnetic field does not show changes in viability or morphology. MDMs were differentiated in the absence or presence PAC-IONs for 5 days and nonexposed or exposed to a 1.5-T MF (MF^-^ and MF^+^, respectively) for 10 min. Photographs were taken with a contrast microscope (40x objective; **A–D)**; then, cells were detached from the plates and stained with DIOC_6_ and PI **(E–H)**. MDMs differentiated w/o PAC-IONs and MF^-^ **(A & E)** w/o PAC-IONs and MF^+^ **(B & F)**, with PAC-IONs and MF^-^ **(C & G)** or with PAC-IONs and MF^+^ **(D & H)** conditions. Figures are one image representative out of n = 5 independent experiments with three replicates each, in which there were no significant differences due to the PAC-IONs or the MF. MDM: Differentiation into mature macrophage; MF: Magnetic field; PAC-ION: Poly(acrylic acid)-coated iron oxide nanoparticle; PI: Propidium iodide.

Although the evidence showed that the PAC-IONs did not compromise the PBMC viability, the MDM viability was studied to verify that cells had not been affected as a result of the high PAC-IONs uptake by their monocytic precursors and the exposure to the MF. MDMs were differentiated in the presence of the PAC-IONs for 5 days and then exposed to the MF for 10 min. Then, the MDM morphology was evaluated by microscopy; additionally, the mitochondrial membrane potential and the cell membrane integrity were evaluated using DIOC_6_ and PI stains, respectively. The morphological characteristics of MDMs did not change after the treatments (Figure 5A–D). Moreover, viable DIOC_6_^high^ MDMs accounted for almost 90% of cells, and MDMs with a damaged cell membrane (PI^+^) were around 10% (Figure 5E–H).

### Accumulation of cytokines in cultures of MDMs differentiated in the presence of PAC-IONs & exposed to MF

**Figure 6.**
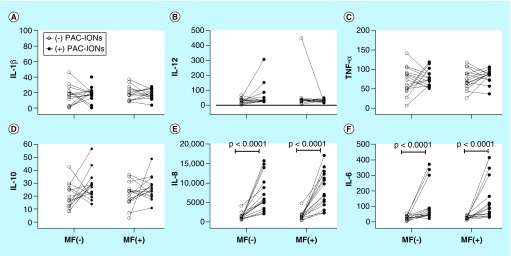
The differentiation into mature macrophages differentiated in the presence of the poly(acrylic acid)-coated iron oxide nanoparticles exhibits increased levels of IL-6 and IL-8 regardless of the exposure to the magnetic field. MDMs were differentiated in the absence (open circles) or presence (filled circles) of PAC-IONs for 5 days and nonexposed or exposed to a 1.5-T MF (MF^-^ and MF^+^, respectively) for 10 min. The levels of cytokines were evaluated by CBA in supernatants of cultures **(A–F)**. The comparisons were made with a two-way ANOVA. n = 5 independent experiments with three replicates each. ANOVA: Analysis of variance; CBA: Cytometric bead array; MDM: Differentiation into mature macrophage; MF: Magnetic field; PAC-ION: Poly(acrylic acid)-coated iron oxide nanoparticle.

The pattern of cytokines accumulated in MDM cultures was studied to verify that it had not been affected as a result of the high PAC-IONs uptake by their monocytic precursors and the exposure to the MF. MDMs were differentiated in the presence of the PAC-IONs for 5 days and exposed to the MF for 10 min. Then, the MDMs were incubated for an additional 6 days, and supernatants were collected for quantifying IL-10, IL-12, TNF-α, IL-1β, IL-8 and IL-6. The transient MF exposure (without PAC-IONs) did not change the levels of cytokines (Figure 6A–F); on the contrary, the exposure to the PAC-IONs (with or without the MF) increased the IL-8 and IL-6 levels (Figure 6E & F). No significant differences were observed in the levels of IL-10 (Figure 6D), IL-12 (Figure 6B), TNF-α (Figure 6C) and IL-1β (Figure 6A). The combined exposure to the PAC-IONs and the MF increased the IL-6 (p < 0.05; Figure 6F), but not the IL-8 (p > 0.05; Figure 6E) levels.

### Accumulation of cytokines in the cocultures of MDMMs (differentiated in the presence of PAC-IONs & MF) with autologous T cells

**Figure 7.**
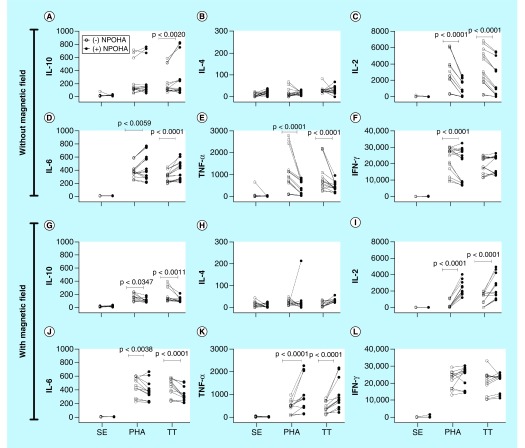
The differentiation into mature macrophages differentiated in the presence of the poly(acrylic acid)-coated iron oxide nanoparticles and exposed to the magnetic field shows a different pattern of cytokines when cocultured with autologous CD3^+^ T cells. MDMs were differentiated in the absence (open circles) or presence (filled circles) of PAC-IONs for 5 days and nonexposed or exposed to a 1.5-T MF for 10 min. Then, MDMs were incubated for 6 days; supernatants were removed, autologous CFSE^+^ CD3^+^ T cells were added (1:2 ratio; MDMs:T cells) and stimulated with PHA or TT for 96 h. The levels of cytokines were evaluated by CBA in supernatants of co-cultures prepared with MDMs that had not been **(A–F)** or had been exposed to the MF **(G–L)**. Comparisons were made with ANOVA II, p-values are shown in the figure; n = 5 independent experiments with three replicates each. ANOVA: Analysis of variance; CBA: Cytometric bead array; MDM: Differentiation into mature macrophage; MF: Magnetic field; PAC-ION: Poly(acrylic acid)-coated iron oxide nanoparticle; PHA: Phytohemagglutinin; TT: Tetanus toxoid.

MDMs differentiated in the presence of the PAC-IONs for 5 days and exposed to the MF for 10 min were incubated for an additional 6 days. Then, supernatants were removed for quantifying cytokines, as described above, and replaced with fresh media containing highly purified autologous CFSE^+^ CD3^+^ T cells (MDM:T-cell ratio = 1:2). The cocultures were stimulated with PHA-M as a positive control for T-cell proliferation or with TT as a conventional antigen for the proliferation of memory T cells. Negative controls for cell proliferation were prepared without any stimulus. After 96-h incubation, supernatants were collected to evaluate the accumulation of Th1/Th2 cytokines: IL-10, IL-4, IL-2, IL-6, TNF-α and IFN-γ. Overall, none of these cytokines increased in the non-stimulated co-cultures (data not shown), and IL-4 levels did not change even in cocultures stimulated with PHA-M and TT (Figure 7B). In comparison with the nonstimulated cocultures, PHA-M and TT induced changes in the levels of IL-10, IL-2, IL-6, TNF-α and IFN-γ (Figure 7). The previous exposure to the PAC-IONs (without the MF) increased the levels of IL-10 in response to TT (Figure 7A), and of IL-6 (Figure 7D) in response to PHA-M and TT. Moreover, the levels of IL-2 (Figure 7C) and TNF-α (Figure 7E) decreased in response to PHA and TT; and, IFN-γ also decreased (Figure 7F) in response to PHA. In the case of previous exposure to the PAC-IONs and the MF, a decrease in the IL-10 (Figure 7G) and IL-6 (Figure 7J) levels were observed in response to PHA and TT. Besides, there was an increase in IL-2 (Figure 7I) and TNF-α (Figure 7K) levels in response to PHA-M and TT, and no differences were found in the accumulation of IFN-γ (Figure 7L) with any of the stimuli.

### Differentiation of MDMs in the presence of PAC-IONs & MF did not affect the proliferation of autologous CD4^+^ or CD8^+^ T cells

**Figure 8.**
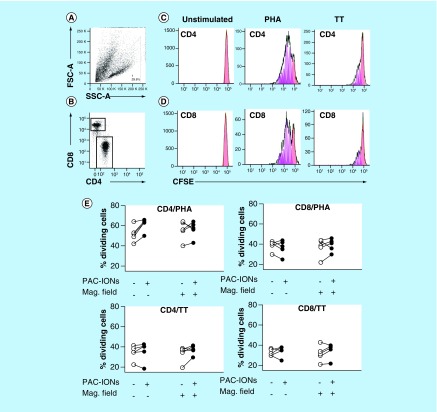
The differentiation into mature macrophages differentiated in the presence of the poly(acrylic acid)-coated iron oxide nanoparticles and exposed to the magnetic field retains the ability to stimulate the proliferation of autologous CD3^+^ T cells. MDMs were differentiated in the absence or presence of the PAC-IONs for 5 days and non-exposed or exposed to a 1.5-T MF for 10 min. Autologous CFSE^+^ CD3^+^ T cells were added (1:2 ratio; MDMs:T cells) and stimulated with PHA or TT for 96 h. CD3^+^ T cells were stained with anti-CD4 and anti-CD8 mAbs. Flow cytometry FSC-A versus SSC-A dot plots for selection of viable lymphocytes **(A)** followed by gating of CD4^+^ and CD8^+^ subpopulations **(B)** to evaluate their proliferation in response to a negative control, PHA and TT by the dilution of CFSE. Histograms from a representative experiment show resting cells (red), dividing cells (pink) and the predicted model of cell proliferation (green line) in the FlowJo software version 7.6.2 (**C**, CD4^+^ and **D**, CD8^+^ T cells). Consolidated data of the percentages of dividing CD4^+^ and CD8^+^ T cells **(E)**. Differences were evaluated with the Wilcoxon test, n = 5, independent experiments. ANOVA: Analysis of variance; CBA: Cytometric bead array; CFSE: Carboxyfluorescein diacetate succinimidyl ester; FSC: Forward scatter; mAb: Monoclonal antibody; MDM: Differentiation into mature macrophage; MF: Magnetic field; PAC-ION: Poly(acrylic acid)-coated iron oxide nanoparticle; PHA: Phytohemagglutinin; SSC: Side scatter; TT: Tetanus toxoid.

MDMs differentiated in the presence of the PAC-IONs and exposed to the MF were cocultured with highly purified CFSE^+^ CD3^+^ T cells and stimulated with PHA-M or TT as previously described. T cells were recovered from the culture, labeled with anti-CD4-PE and anti-CD8-eFluor mAbs, and acquired in a flow cytometer to evaluate the cell proliferation. SSC-A^low^ dead cells were excluded from the analysis in an FSC-A versus SSC-A dot plot (Figure 8A; they were also DAPI^+^ cells, data not shown). CD4^+^ and CD8^+^ T cells were gated (Figure 8B) to analyze the dilution of CFSE in the proliferating cells (Figure 8C & D). Overall, the cell proliferation, the division indexes (data not shown) and the percentages of dividing T cells in response to TT or PHA-M (Figure 8E) did not change in co-cultures prepared with MDMs previously exposed to the PAC-IONs and/or the MF. Although the percentage of dividing T cells increased in the cocultures with MDMs previously exposed to PAC-IONs, this result was not statistically significant.

### CD14^+^CD16^+^ monocytes showed a higher PAC-ION uptake than the CD14^+^CD16^-^ subset

**Figure 9.**
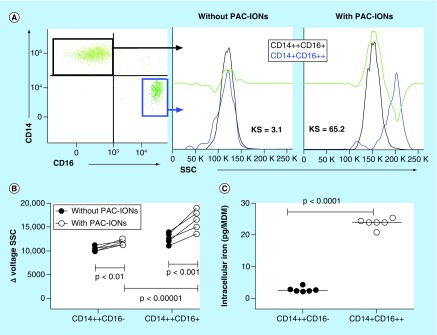
Nonclassical monocytes have a higher uptake of poly(acrylic acid)-coated iron oxide nanoparticles. Whole-blood samples were treated with PAC-IONs for 1.5 h and stained with anti-CD45-PE-Cy7, anti-CD14-PE, anti-CD16 V450 and anti-HLA-DR-FITC. Flow cytometer dot plots show the distribution of classical, nonclassical and intermediate monocytes, then overlaid histograms through the K–S Goodness-of-Fit test compared the granularity in the absence (left) or in the presence of PAC-IONs (right). The changes in the granularity for classical and nonclassical monocytes were compared by test in the FlowJo program version 7.6.2. The consolidated information of five independent experiments is shown in **(B)**. MDMs were differentiated from CD14^+^CD16^-^ (CD14^+^) or CD14^+^CD16^+^ (CD16^+^) monocytes in the absence or presence of PAC-IONs for 5 days **(C)**; then, MDMs were lysed and acid-digested for quantifying the intracellular iron content by AAS. The comparison was made with the Wilcoxon test, n = 4, independent experiments. AAS: Atomic absorption spectrometry; KS: Kolmogorov–Smirnov; MDM: Differentiation into mature macrophage; PAC-ION: Poly(acrylic acid)-coated iron oxide nanoparticle; SSC: Side scatter.

In order to establish if monocyte subsets had a different ability for PAC-ION uptake, whole-blood samples were exposed or not to the PAC-IONs only for 1.5 h (this shorter incubation time was set because the surface CD14 and CD16 expression in monocytes decreased during more extended periods). Then, cells were stained with anti-CD45-PeCy7, anti-CD14-PE, anti-CD16-BV450 and anti-HLA-DR-FITC mAbs to be analyzed by flow cytometry. In the first place, CD14^+^^+^CD16^-^ and CD14^+^CD16^+^^+^ monocyte subsets were identified; then, the cell granularity was evaluated in each cell subpopulation from cultures previously exposed or not to the PAC-IONs; and finally, the Overton subtraction was used for comparing the granularity of the cell subsets (Figure 9A). In the absence of the PAC-IONs, the Kolmogorov–Smirnov test between the two monocyte subsets was about 3.2%, but in the presence of PAC-IONs, the difference was 65%. The Δvoltage SSC for cells before and after exposure to the PAC-IONs showed that the CD14^+^CD16^+^^+^ monocytes had the higher PAC-ION uptake (p ≤ 0.00001; Figure 9B). Additionally, purified CD14^+^CD16^-^ and CD14^+^CD16^+^ monocytes were allowed to differentiate in the presence of the PAC-IONs for 5 days. Then, cells were lysed to quantify the intracellular iron content by AAS. MDMs derived from the CD14^+^CD16^+^ monocytes showed an iron content around ten-times higher compared with MDMs derived from the CD14^+^CD16^-^ subset (p < 0.0001; Figure 9C).

## Discussion

Nanomedicine has implemented the use of materials in the nanoscale range to improve different features of clinical processes, from diagnosis to treatment. Nanoparticles have been used as vehicles, containers for drug encapsulation, anchor points for proteins, enzymes and even iRNA, and contrast agents, for different purposes.

The contrast agents used in nanomedicine are coated or uncoated metal nanoparticles. The core can be made of gold, silver or magnetic oxides (copper, iron, cobalt and titanium). The latter allow the detection of a magnetic response when the nanomaterials are exposed to an MF. Previous studies have shown that iron oxides coated with polymers have a reasonable degree of biocompatibility [46–49]. For this reason, PAC-IONs were used in this study as a putative interface to interact with monocytes. This study evaluated some of the possible effects of these nanoparticles on PBMCs and MPs, taking into account that the PAC-IONs are a putative interface to interact with MPs through scavenger receptors [50].

### Effect of the PAC-IONs on the viability of PBMCs

The results showed that the PAC-IONs and the MF do not affect either the cell membrane integrity or the mitochondrial membrane potential of PBMCs after 12 h of exposure; besides, they do not induce DNA strand breaks, apoptosis or changes in the proliferation of PBMCs stimulated with PHA or TT. These results agree with data published in the literature that reported that human T cells exposed to PAC-IONs with a diameter of 10.1 nm did not undergo alterations in either chromosomes or cell proliferation [47]. However, another study in a murine model found that cardiac tissues exposed to polyethylene glycol-coated IONs with an average diameter of 5 nm showed an increased production of intracellular reactive oxygen species (ROS), as well as DNA damage, evidenced by the COMETA assay [51]. Another study found that the treatment of K562 cells with daunorubicin (inhibitor of topoisomerases) plus Fe_3_O_4_ nanoparticles 30 nm average diameter enhanced the nuclear fragmentation induced by the drug through the caspase-8/PARP pathway [52]. Overall, these studies highlight the relevance of the particle size rather than the type of coating in the activation of different pathways able to induce cell damage.

### Selective PAC-IONs uptake by the leukocyte subpopulations

Because the PAC-IONs did not affect the PBMC viability, the selective particle uptake by different leukocyte subsets was evaluated through the changes in cell granularity (SSC-A) by flow cytometry, as previously reported [53,54]. The highest PAC-ION uptake by monocytes was verified by quantification of the intracellular iron content by AAS, as previously reported by other authors [45]. The MDMs cultured in the presence of PAC-IONs had an iron content seven-times greater (Figure 4C) than the amount observed in MDMs differentiated without the nanoparticles. This finding confirmed that the change in the monocyte granularity was related to the PAC-IONs internalization.

Cell granularity as an indicator of particle uptake has been used by other authors who reported that iron levels could be equivalent to 50% of the uptake observed in the present study [56]. This finding could be attributed to the different concentration of nanoparticles; in fact, they used 100 μg/ml while the present study used 32 μg/ml in all cases. On the other hand, polyacrylate could have given selectivity for the PAC-IONs to monocytes; however, there is not enough evidence to determine the cause of the higher nanoparticle uptake observed in the present study. To date, there are no reports about the mechanism of recognition involved in the PAC-IONs internalization by monocytes; however, the selectivity for monocytes is attributed to the Z potential. This factor allows the interaction with receptors that can bind negatively charged particles, that can enter monocytes via the MARCO scavenger receptor [55]. Previous reports have proposed that the potential interaction between nanoparticles and scavenger receptors could be related to the sodium polyacrylate conjugated on the surface of liposomes [50]. Moreover, owing to the PAC-IONs’ sodium polyacrylate coat, the nanoparticle surface is enriched in carboxyl groups, and can interact with the CD36 scavenger receptor [50].

### Effect of the PAC-IONs & the MF on the MDM viability

According to the present study, the PAC-IONs had a higher selectivity for monocytes; therefore, the following analysis was focused on this leukocyte subpopulation. Viability, morphology and cytokine production were evaluated in MDMs differentiated in the presence of the PAC-IONs and the MF.

MDMs differentiated in the presence of the PAC-IONs and/or the MF did not show any difference in the DIOC_6_ uptake compared with MDMs differentiated in the absence of those treatments. This observation indicated that neither the presence of the PAC-IONs nor the exposure to the MF affected the MDM viability. These findings agree with a previous work in which the monocyte viability was not affected by four independent preparations of polymeric dextran-coating IONs [45].

In addition to cell viability, it was relevant to evaluate the events involved in the function of the MPs, including adherence, differentiation and cytokine production. To this purpose, MDMs differentiated in the presence of the PAC-IONs and exposed to the MF were visualized under phase-contrast microscopy. The images showed that the adherence to the plate and the typical cytoplasmic projections were similar to those observed in the control cultures.

Other authors exposed monocytes to 100 μg Fe/ml of dextran coated-IONs for 2 h and found no effect on the CD11b expression [45]. The fact that the PAC-IONs and the MF did not change the protein content suggested that cellular adhesion had not been altered, and it was considered that the number of adherent cells did not change during the process of differentiation in the presence or absence of the PAC-IONs. The exposure of U937 promonocytes to a 6-mT MF together with 12-0-tetradecanoyl-13-phorbol-acetate, a differentiation inducer, altered the cell morphology, decreased the number of adherent cells and affected the differentiation of the cell line [56].

### Effect of the PAC-IONs & the MF in the production of cytokines in MDM cultures & cocultures of MDMs with autologous T cells

To explore functional changes in MDMs differentiated in the presence of the PAC-IONs and the MF, two parameters were evaluated: the levels of cytokines in the cultures of MDMs and the interaction of MDMs with autologous T cells.

#### Accumulation of cytokines in MDM cultures

Cytokines were measured in supernatants from MDMs differentiated in the presence of the PAC-IONs and exposed to the MF. IL-6 and IL-8 increased in the MDM cultures differentiated in the presence of PAC-IONs regardless of the exposure to the MF. Similar results were reported by some authors who argued that the phenomenon could be explained by an increase in the ROS levels [57,58]. Although ROS production was not evaluated in the present study, it is possible to suppose that mitochondria were not compromised because the DIOC_6_ uptake was similar in cultures exposed or not to the treatments. Neutrophils increase the ROS production in response to polyacrylate-coated nanoparticles, in a mechanism dependent on the NADPH oxidase system [59]. In the experimental model here presented, a test for a specific evaluation of ROS production in response to PAC-IONs would be convenient. In another study, the treatment of whole blood with PAC-IONs induced the production of IL-1β, TNF-α, IL-6, IL- 8, IFN-γ and IL-10 [46], a finding partly similar to results here described where only increased levels of IL-6 and IL-8 were observed. Other authors found that proinflammatory cytokines (TNF-α, IL-1β, IL-6) did not increase in cultures of the human monocytic cell line THP-1 exposed to polymeric chitosan DNA-encapsulating nanoparticles [60], suggesting a possible role for the type of coating.

Considering that the MDMs were not only treated with the PAC-IONs but also exposed to the MF, it is necessary to deliberate if this treatment could alter the production of cytokines. It is known that MFs can affect the channels, such as the voltage-gated calcium one, which in turn participates in the proper functioning of the potassium channels [61,62]. These latter ones are involved in the production of some cytokines [56,63], an event activated as soon as the macrophages begin to adhere in response to IL-2 or IL-6 [63]. These observations could partly explain the increased levels of IL-8 and IL-6 in the MDM cultures differentiated in the presence of the PAC-IONs and exposed to the MF.

#### Accumulation of cytokines in cocultures of MDMs & autologous T cells

A stepwise system for coculture of MDMs and T cells was prepared. In the first stage, monocytes were isolated, treated with the PAC-IONs and the MF, and allowed to differentiate into MDMs for 5 days. In the second stage, the MDMs were washed and incubated for 5 days before being cocultured with purified autologous CD3^+^ T cells (1:2 cell ratio) and treated with PHA or TT for an additional 96 h. In the absence of the PAC-IONs and/or the MF, the cytokine accumulation was similar. In the presence of the PAC-IONs and/or the MF, without PHA or TT, there was no effect either. In contrast, in cocultures prepared with MDMs differentiated in the presence of the PAC-IONs but non-exposed to the MF, and stimulated with PHA or TT, the levels of IL-2, TNF-α and IFN-γ decreased and those of IL-10 and IL-6 increased. An inversion of this effect was observed for MDMs differentiated in the presence of the PAC-IONs and the MF.

The effect of the interaction between the PAC-IONs and the MF on the cytokine accumulation was evaluated up to the second degree with an ANOVA II. No significant interactions were observed for any of the cytokines analyzed. It is important to note that IL-6 accumulation, observed even in nonstimulated cultures, was not evidenced after the addition of T cells, whereas the lymphocytes were added after washing the cultures. Possibly, the consumption of this cytokine by the cellular interactions did not allow it to be detected in the culture supernatants. Besides, the treatments could affect other interactions between MPs and T cells that were not detectable by the approach used.

### Proliferation of memory T cells co-cultured with MDMs differentiated in the presence of the PAC-IONs & exposed to the MF

Other functions of MDMs, such as their role in the antigen-dependent proliferation of CD4^+^ and CD8^+^ T cells seems to not affect the interactions with PAC-IONs, suggesting that the nanoparticles did not functionally compromise the monocyte differentiation into macrophages. Besides, this finding directly supports our previous observation that HLA-DR and CD80 (involved in antigen presentation and stimulation of T-cell proliferation) are not affected by the differentiation of MDMs in the presence of the PAC-IONs; moreover, it provides indirect evidence of a similar effect for the MF.

PHA and TT require different pathways to induce the proliferation of T cells. While PHA induces a nonspecific polyclonal proliferation that does not require antigen presentation, the TT does. However, the pattern of cytokines accumulated in the cocultures changed regardless of being stimulated with PHA or TT. This result suggested that the altered pattern of cytokines could not be attributed to the MDM–lymphocyte interaction but the response of MDMs to the PAC-IONs and the MF.

### Evaluation of the PAC-ION uptake by monocyte subpopulations

When the monocyte subpopulations were cultured with the PAC-IONs, the CD14^+^CD16^+^ subset showed an increased granularity (data not shown). Besides, higher iron content was found in the lysates prepared from macrophages derived from the CD14^+^CD16^+^ monocytes and differentiated in the presence of the PAC-IONs. These MDMs had the highest PAC-ION uptake, 20–25 pg of Fe/cell versus 2–4 pg of Fe/cell by the macrophages derived from the classical CD14^+^CD16^-^ monocytes. This result contrasts with a previous study [45] in which CD14^+^CD16^-^ monocytes showed the highest uptake of polymer-coated IONs, 5–30 pg Fe/cell versus 2–10 pg Fe/cell for the CD14^+^CD16^++^ monocytes [45].

Considering that PAC-IONs can be used for drug-anchoring, an interesting perspective would be the concomitant use of PAC-IONs and rifampicin in animal models with tuberculosis to evaluate the possibility of reducing the antibiotic efflux. This strategy was used in a model with *Mycobacterium smegmatis*, where it increased the effectiveness of the antibiotic treatment. Also, this strategy could allow the location of infiltrating monocytes in the pulmonary tissue [64].

Considering that the PAC-IONs’ sodium polyacrylate coating seems to define their selectivity for monocytes, it would be essential to determine the way this interaction occurs in the translation into specific applications. The particles have a carboxyl-enriched surface available for the union of different molecules, such as iRNA, that could be useful for other purposes. The PAC-ION relevance in drug-anchoring has been reported in the modulation of osteoclasts by blocking the expression of RANK with shRNA [65]. In a murine model of arthritis, bullet monocytes can be prepolarized before they arrive at the joint to avoid their differentiation into osteoclasts [66]. Other different diseases, such as chronic obstructive pulmonary disease or asthma [67], cigarette smoking with asthma or scleroderma-associated fibrosing alveolitis [68,69], could be studied with nanoparticles, precluding the requirement of invasive biopsies; additionally, they could be used to perform drug delivery. Bierry *et al.* [70] used MRI with superparamagnetic iron oxide (SPIO) gadolinium to identify the macrophages present in injured tissues of patients with infectious vertebral osteomyelitis and degenerative disk-related inflammatory endplates [70]. In knees of rabbits that had been presensitized with methylated bovine serum albumin and unilateral arthritis was induced by means of intra-articular injection of the same antigen, imaging at 1.5 T and 24 h after the contrast agent administration could evidence the ultrasmall superparamagnetic iron oxide (USPIO) uptake by phagocytic-active macrophages in the synovium of all the arthritic knees [71].

Putting the results in perspective, if the PAC-IONs were considered for use as a contrast agent, it would be crucial to study their biodistribution and pharmacokinetics in animal models. Although these studies are beyond our laboratory feasibilities, it is important to highlight that it is feasible to selectively track phagocytes as reported by Tracke *et al.* [72], who developed an approach with beads 0.5–1 m that were preferentially internalized by nonclassical monocytes [72]. Then, the authors considered that it was possible to selectively label mouse monocyte subsets *in vivo* without further perturbations to the animal; it is worth noting that these treatments had minimal impact on the physiological cell function [73], which is in agreement with our findings.

## Conclusion & future perspective

PAC-IONs can selectively interact with MPs, particularly with the nonclassical ones, without affecting their MDMs. Moreover, the exposure of these monocytes to the PAC-IONs and the MF do not induce cell damage or compromise their function as antigen-presenting cells, evaluated in terms of cytokine production and induction of the activation and proliferation of T cells in response to standard antigens. The fact that PAC-IONs seem to be more selective for nonclassical monocytes makes them a very attractive and clinically relevant tool to study these cells by MRI and for specific purposes to control monocyte differentiation, polarization and interaction with endothelial cells. It is hoped that very soon PAC-IONs will allow identification of the location of inflammatory monocytes without requiring invasive strategies.

**Summary points**

- Poly(acrylic acid)-coated iron oxide nanoparticles (PAC-IONs) are selectively internalized by monocytes.
- Monocytes differentiate into macrophages (MDMs) in the presence of PAC-IONs without undergoing cell damage.
- The magnetic field (MF) does not alter the MDM viability even in the presence of PAC-IONs.
- MDMs differentiated in the presence of PAC-IONs and exposed to MFs do not show a significant compromise in the accumulation of cytokines.
- MDMs differentiated in the presence of PAC-IONs and exposed to MFs can activate T cells properly.
- The selective PAC-ION uptake by nonclassical monocytes makes these cells an attractive tool to target diseases with a chronic inflammatory process.
